# A Cortico- Basal Ganglia Model for choosing an optimal rehabilitation strategy in Hemiparetic Stroke

**DOI:** 10.1038/s41598-019-49670-4

**Published:** 2019-09-17

**Authors:** Rukhmani Narayanamurthy, Samyukta Jayakumar, Sundari Elango, Vignesh Muralidharan, V. Srinivasa Chakravarthy

**Affiliations:** 1 Bhupat and Jyoti Mehta School of Biosciences, Department of Biotechnology, Indian Institute of Technology Madras, Chennai, India; 2 Department of Psychology, University of California, San Diego, USA

**Keywords:** Network models, Stroke

## Abstract

To facilitate the selection of an optimal therapy for a stroke patient with upper extremity hemiparesis, we propose a cortico-basal ganglia model capable of performing reaching tasks under normal and stroke conditions. The model contains two hemispherical systems, each organized into an outer sensory-motor cortical loop and an inner basal ganglia (BG) loop, controlling their respective hands. The model is trained to simulate two therapeutic approaches: the constraint induced movement therapy (CIMT) in which the intact is arrested, and Bimanual Reaching in which the movements of the intact arm are found to aid the affected arm. Which of these apparently mutually conflicting approaches is right for a given patient? Based on our study on the effect of lesion size on arm performance, we hypothesize that the choice of the therapy depends on the lesion size. Whereas bimanual reaching is more suitable for smaller lesion size, CIMT is preferred in case of larger lesion sizes. By virtue of the model’s ability to capture the experimental results effectively, we believe that it can serve as a benchmark for the development and testing of various rehabilitation strategies for stroke.

## Introduction

Stroke is considered to be one of the leading causes of disability and mortality worldwide. It generally manifests itself as an upper extremity dysfunction with 80% patients suffering from it acutely and 40% chronically^1^. Sensory and motor deficits resulting from unilateral stroke include difficulty in performing common activities like reaching, grasping and picking up objects. Functionally, hemiparesis is one of the common motor impairments associated with unilateral stroke, after which a more obvious deterioration in the performance of the contralateral arm is observed^2^. Recovery following motor rehabilitation post-stroke primarily depends on the initial severity of paresis^3^ and degree of loss in functionality. It has been reported that the prognosis is good when patients are treated within the first three months following stroke^4^. Nevertheless, reports show upper extremity recovery ensuing several years after stroke^5,6^. Hence it has been suggested that progress in functional outcome may be due to neurological repair through cortical reorganization or compensatory mechanisms^7^.

The ultimate objective of a wide variety of existing rehabilitation protocols like virtual reality based rehabilitation^8,9^, music therapy^10^, mirror therapy^11^, motor imagery^12,13^, motor imitation^14^, movement observation^15^, transcranial magnetic stimulation (rTMS)^16^, unilateral muscle strengthening exercises^17,18^, motor skill learning^19^, bimanual and unimanual training^20^ (e.g., Constraint Induced Movement Therapy (CIMT)), is to enable patients recover from weakness and improve functionality of the arm. However, the problem of determining the best approach for a given stroke patient is inconclusive due to inherent contradictions in some of the existing rehabilitation approaches^21^. For instance, CIMT is primarily based on the repetitive use of the affected arm by restraining the healthy arm^19^ while bimanual training pairs the healthy arm with the paretic arm to increase the chances of recovery^22^.

A wide variety of computational models/frameworks have been proposed for motor control by the human motor system. The model of Chen *et al*.^23^ consists of a motor cortex (MC) that controls a 2-link arm through motor neurons and a proprioceptive cortex that provides feedback to the MC. The movement of the arm is controlled by stimulating different nodes of the MC and maps are formed with the help of unsupervised learning. However due to the absence of basal ganglia (BG), the arm is unable to make reaching movements to a specific target. The model proposed by Han *et al*.^24^ is bimanual and the choice between using either of the two hands is made with the help of an Action Choice Module. This module compares the value of making the movement with either hand and chooses the hand with the highest value. Reinforcement learning is used by the model to achieve this. In the model proposed here, comparison is made between making and not making a movement similar to that performed by direct vs indirect pathway of the BG. On similar lines, Takiyama *et al*.^25^ investigated the potential mechanisms that contribute towards the efficacy of bimanual movements in stroke recovery from a computational perspective. Although it gives an insight into the neural mechanism that drives cortical reorganization following bimanual rehabilitation, it does not account for the various cortical and subcortical structures involved in motor control. Other models like the ones proposed by Hidaka *et al*.^26^ and Casadio *et al*.^27^ were specifically developed for stroke and are based on real patient data. However, these models were developed for specific treatment regimes (robot-assistance therapy and CIMT respectively) thereby restricting the scope of incorporating other therapies into their models. A more thorough review of the various computational models of stroke and rehabilitation can be found in Reinkensmeyer *et al*.^28^.

In this paper we use computational modelling to study two common rehabilitation strategies – bimanual training and CIMT. While bimanual training recommends use of both hands since the normal hand aids in the rehabilitation of the paretic hand, CIMT posits that the normal hand must be arrested to permit optimal rehabilitation of the paretic hand. The two strategies are obviously mutually contradictory. Which strategy is more suitable in a given situation? We address this question using an elaborate computational model of the cortico-basal ganglia network.

Our model is designed to perform both unimanual and bimanual reaching under different task conditions. The model consists of an outer cortical loop and an inner basal ganglia loop. Performance is evaluated in terms of peak resultant velocity (PRV) and reaching error. The entire system drives a two-link arm model engaged in targeted reaching movements. The basal ganglia loop primarily drives motor learning with the control gradually passed on from the basal ganglia (BG) to the motor cortex (MC) as learning progresses. Two copies of the entire system, appropriately coupled, is used to simulate bimanual reaching. The model is able to explain reaching behaviour under normal and hemiparetic stroke conditions and is also able to capture key results from the bimanual reaching experiments of Rose and Winstein^29^ where it is shown that bimanual reaching tasks are beneficial in reviving the activity of the affected limb post stroke. The model is also operated under CIMT conditions. A comparison of the effect of the two apparently contradictory strategies on model performance pointed to an interesting resolution: bimanual reaching is found to be more beneficial for smaller lesion sizes whereas CIMT is more effective for bigger lesion sizes.

The outline of the paper is as follows. Section 4 describes the model equations and task setup. Section 2 describes the model results related to unimanual and bimanual reaching. A comparison of recovery from stroke following CIMT and bimanual reaching is also presented. Section 3 presents a discussion of the results and scope for future work.

## Results

### Simulating intra-cortical connectivity

The bimanual aspect of the model has its seed at the level of the respective motor cortices of the two arms. The MCs communicate by means of a “coupling factor (*ε*)” where, the product of *ε* and MC activity (${\varepsilon }_{RIGHT/LEFT}\times {G}_{MC}^{RIGHT/LEFT}(t)$) of the ipsilateral hemisphere is added with the input current to the MC CANN (${I}_{MC}^{RIGHT/LEFT}(t)$) of the contralateral hemisphere. Based on Eq. (17) (under Methods), this coupling is mathematically represented by,

1 $${I}_{MC}^{RIGHT}(t)={A}_{PC}{G}_{PC}^{RIGHT}(t)+{A}_{BG}{G}_{BG}^{RIGHT}(t)+{A}_{PFC}{G}_{PFC}^{RIGHT}(t)+{\varepsilon }_{RIGHT}{G}_{MC}^{LEFT}(t)$$

2 $${I}_{MC}^{LEFT}(t)={A}_{PC}{G}_{PC}^{LEFT}(t)+{A}_{BG}{G}_{BG}^{LEFT}(t)+{A}_{PFC}{G}_{PFC}^{LEFT}(t)+{\varepsilon }_{LEFT}{G}_{MC}^{RIGHT}(t)$$

Based on the coupling strengths, the reaching task conditions could be categorized into:

- **Unimanual condition:** Unimanual movement is implemented in the model under the assumption that the resting arm has limited influence over the moving arm. For instance, when the right arm moves, the value of *ε*_*RIGHT*_ in Eq. (1) will be low, signifying the minimal effect of the ${G}_{MC}^{LEFT}(t)$ on the right arm and vice versa.
- **Bimanual condition:** Here the coupling values of *ε*_*RIGHT*_ and *ε*_*LEFT*_ are non-zero and are optimized to fit the data.
- **Constraint induced movement:** Here the coupling values have the same range as in bimanual condition. However in this case, unlike bimanual, ${G}_{MC}^{LEFT}(t)$ remains constant denoting the dormant nature of the constrained, unaffected (left) arm.

The velocity profiles (refer Supplementary Material) of the aforementioned task conditions when performed in the absence of lesion, serve as control data for comparison with performance of the model post-stroke.

### Simulating Hemiparetic Stroke in the model

To simulate and study hemiparetic stroke, we incorporated “lesion” of size n × n in the right MC of our model by suppressing the activity of a fixed number of nodes in g_MC_ of the CANN as follows:

3 $${g}_{MC,i,j}(t)=0.01{g}_{MC,i,j}(t)$$

where,

g_MC_ is the state of neurons in MC CANN at time ‘t’.

(i, j) corresponds to a 2D array in g_MC_ that is used to define the lesion in MC.

a and b define the location of the lesion in the MC.

### Performance of the arm post-stroke simulation

**Figure 1:**
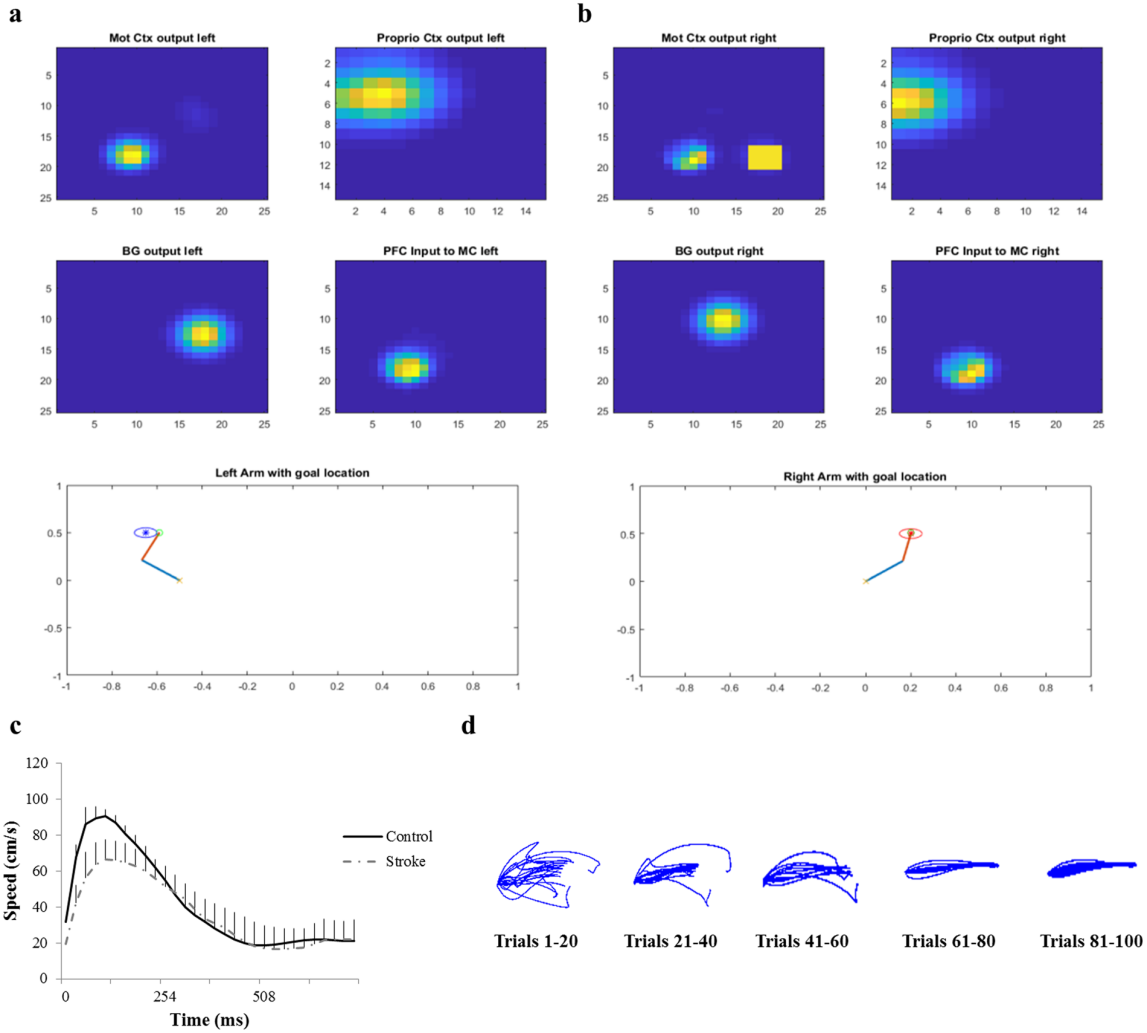
Reaching behaviour in stroke. (**a**,**b**) The network output of the left and right arm while performing the reaching task independently and the activities of multiples areas in the model. (**c**) The velocity profile of the right arm (now paretic) during a reach. The yellow square in MC right denotes the presence of lesion. (**d**) The end effector trajectories of the paretic arm obtained for reaching a single target across trials as the learning of the PFC to MC connections (W_PFC→MC_) takes place.

To test changes in the kinematics of the arm after introducing lesion, we estimated the velocity profile of the affected arm while it was performing a reaching task. The network was trained to reach a single target followed by introduction of lesion in the corresponding MC. It can be noticed from Fig. 1c that there is a significant decrease in the velocity of the affected arm when compared to its normal counterpart.

### Training the outer motor cortical loop under lesion conditions

#### Retraining PC to MC connection

Once a lesion is introduced, PC to MC connection is retrained such that the winner node corresponding to the end effector position of the arm is picked from the neighbouring nodes that lie outside the lesion area. The winner node is the one whose weight is the least distant from the given input vector. Also, instead of random initialization of SOM weights, weights trained under normal conditions are used to retrain the connections between PC and MC.

#### Retraining MC to MN Weights

MC to MN weights are retrained in order to maintain consistency with respect to retraining the cortical loop once a lesion is introduced. Therefore, the weight update is given as,

4 $$d{W}_{MC\to MN}={\eta }_{MC\to MN}({\varphi }^{MN}(t)-{\varphi }^{MN}(t+1))$$

5 $${W}_{MC\to MN}={W}_{MC\to MN}+d{W}_{MC\to MN}$$

where,

${\eta }_{MC\to MN}$ is the learning rate

$d{W}_{MC\to MN}$ is the weight update

**Figure 2:**
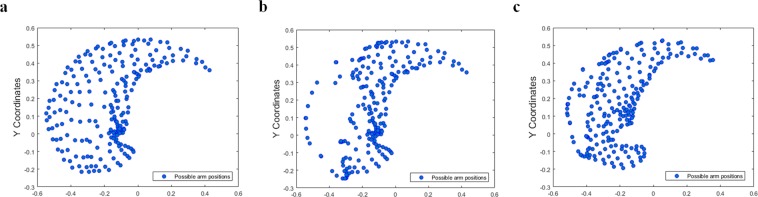
Mapping of the end effector positions approximated by the network. All possible end effector positions of the arm in the given workspace are mapped under three different conditions **(a)** normal(control) condition **(b)** stroke condition and **(c)** retraining the cortical loop post stroke.

${\varphi }^{MN}(t)$ and ${\varphi }^{MN}(t+1)$ are MN outputs computed from motor cortical activities collected at t^th^ and t + 1^th^ time steps when the arm is closest to its goal. The recovery of the lost end effector positions in the workspace after retraining the cortical loop is as shown in Fig. 2c.

### Experimental study – Rose and Winstein (2004)

Rose and Winstein^29^ (2004) investigated the feasibility of bimanual training protocol for post-stroke rehabilitation. They tested the performance of the upper extremities under unimanual and bimanual conditions in three different reaching paradigms. A total of 30 stroke patients, alongside 30 healthy people (controls), were involved in the study. The participants were asked to reach forward rapidly and aim with one hand (unimanual) or both hands (bimanual) and hit switches mounted on LED targets in response to an LED signal.

Their initial study examined a spatially symmetric forward aiming movement of the arms. They found that, in unimanual condition, the non-paretic arm exhibited a higher Peak Resultant Velocity (PRV), than in bimanual condition, where it was paired with the paretic arm. On the contrary, the paretic arm exhibited a higher PRV when it functioned bimanually as opposed to unimanual condition. Interestingly, this discrepancy was observed only in stroke patients and not in controls.

**Figure 3:**
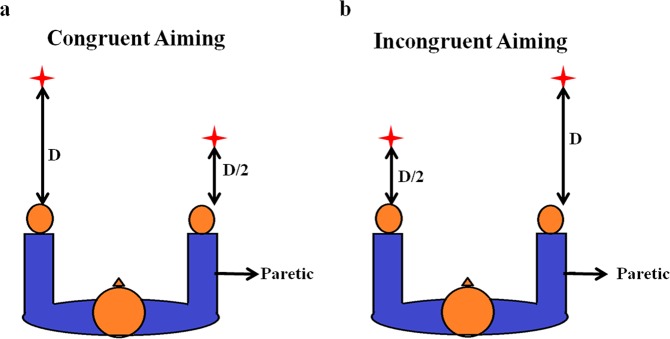
Asymmetric target aiming. (**a**,**b**) Congruent and Incongruent aiming setups in Rose and Winstein’s experiment. ‘D’ refers to the aiming distance. In congruent condition, target for the paretic arm was located at half the distance of target location of the normal arm. In incongruent aiming, the paretic arm had the farther target to reach.

Rose and Winstein extended their study by inducing spatial disparity in reaching distances which would require co-ordination among both arms. Similar to their previous case, the participants had to aim and hit switches in response to an LED signal, only now the arms moved to separate targets. This experiment was classified into two based on the level of difficulty of the task assigned to paretic arm. The first type was called “congruent aiming”, where the paretic arm moved to a “near” target and the non-paretic arm moved to a “far” target while the second type known as “incongruent aiming” followed the converse of the congruent task setup (Fig. 3).

The “near” target is placed at 50% of the distance of the “far” target as shown in Fig. 3. It was observed that the non-paretic limb exhibited a prolonged movement execution time accompanied by a decrease in its PRV in bimanual condition, than that in unimanual condition. Also, there was an increase in the paretic limb PRV in bimanual condition when compared to unimanual condition. However, the enhanced paretic limb PRV was seen only with respect to incongruent aiming which further led them to compare paretic limb PRV in unimanual conditions for the near and far aiming tasks to verify if aiming distance alone facilitated such an increasefacilitated such an increase^29^.

The paretic limb PRV was similar in both unimanual aiming conditions thereby suggesting that both paretic aiming distance and constraints of bimanual coordination are prerequisites to improve performance of the paretic arm.

### Model performance on the reaching tasks

#### Symmetric Aiming

**Figure 4:**
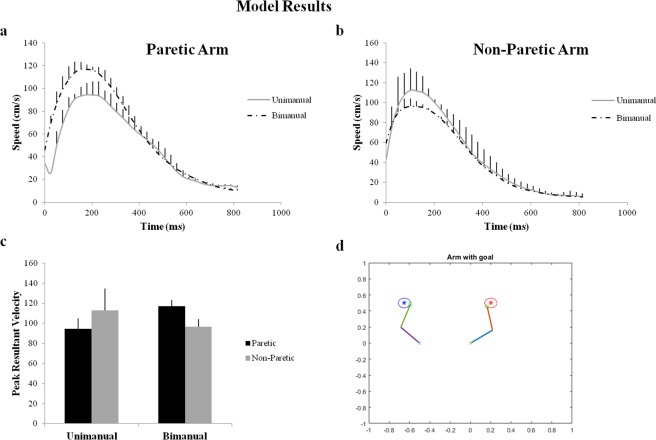
Model performance in symmetric aiming. (**a**,**b**) Velocity profiles of the paretic and the non-paretic arm under unimanual and bimanual conditions obtained from the Model. (**c**) The bar graph of the Peak resultant velocities (from model). (**d**) Snapshot of the simulation of the arms reaching the targets in symmetric aiming task.

We simulated the symmetric aiming task in the model by providing each arm with its respective target, at spatially symmetric locations as shown in Fig. 4d. In the first case, the arms are allowed to perform the reaching under unimanual aiming conditions where ε = 0. This is followed by testing the arm under bimanual conditions using both inhibitory and excitatory coupling (ε < 0 and ε > 0). For each arm, its velocity at every instant in time is recorded and averaged to find the maxima of the velocity profile which is referred to as the PRV.

It is observed that under unimanual conditions, the non-paretic arm (left arm in the model) shows a greater PRV than the paretic arm (right arm in the model). Similarly, it is found that during bimanual training of the arms to accomplish the target, the paretic arm showed a significant increase in its PRV whereas the non-paretic arm showed a decrease in its PRV when compared to unimanual training as shown in Fig. 4a,b. This was achieved by using the coupling factor where there is excitatory influence from the paretic to the non-paretic arm and inhibitory influence from non-paretic to the paretic arm.

#### Congruent and Incongruent Aiming

**Figure 5:**
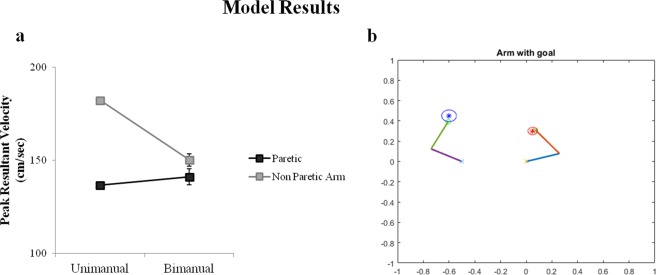
Model performance in congruent aiming. (**a**) Peak resultant velocities of the paretic and the non-paretic arm under unimanual and bimanual conditions obtained from model (**b**) Snapshot of the simulation of the arms reaching the targets in congruent aiming task.

The second experiment conducted by Rose and Winstein required movement of the upper extremities to “near” and “far” targets. Based on this spatial disparity, the tasks were divided into congruent and incongruent aiming tasks. In congruent aiming, the paretic arm had a near target and hence an easy task to accomplish, whereas the non-paretic arm had a far target to reach. The near target is placed at 50% of the distance of the far target as shown in Fig. 5b. We simulated this task setup in the exact same manner and allowed the model to perform the task. The PRVs of both the arms are recorded under unimanual and bimanual conditions. It is found that under unimanual conditions, the non-paretic arm had a higher PRV than the paretic arm with, no significant change in the PRV of the paretic arm in the bimanual case. However a decrease in the velocity of the non-paretic arm is observed when it is paired with the paretic arm (i.e. bimanual) (Fig. 5a).

**Figure 6:**
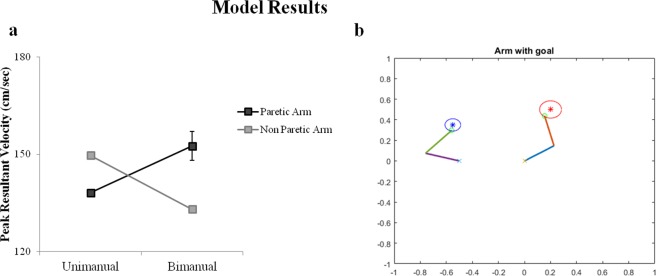
Model performance in incongruent aiming. (**a**) Peak resultant velocities of the paretic and the non-paretic arm under unimanual and bimanual conditions obtained from model (**b**) Snapshot of the simulation of the arms reaching the targets in incongruent aiming task.

The incongruent aiming task observes the converse experimental setup as that of the congruent aiming task. Here, the paretic arm (right) has a far target (difficult task) to accomplish whereas the non-paretic arm (left) has a near target to accomplish as shown in Fig. 6b. A similar recording of the PRV is performed as mentioned in the earlier studies. It is found that the PRV of the non-paretic arm is higher than the paretic arm under unimanual conditions. But in the bimanual conditions, the paretic arm shows a significant increase in its velocity whereas the velocity of the non-paretic arm decreased greatly as shown in the graph (Fig. 6a). The results obtained in all the three aforementioned task setups follow the same trend as those obtained by Rose and Winstein^29^.

### Aiming conditions Vs lesion size

In this part, we study the effects of different lesion sizes of the MC on reaching behaviour. One of the several factors inducing a change in reaching performance could be the extent of damage in the corresponding hemisphere after stroke. The model provides a framework to test this effect and also serves as a tool to compare different interventions currently available to find the best possible one for patients in order to help them regain control. It becomes important to provide a forum that can reconcile different strategies since most of them are inconsistent with each other in relation to their techniques.

Here, we record the reaching error of the arm i.e. the minimum distance of the arm from its target over lesion sizes starting from 1 × 1 to 7 × 7 for CIMT, unimanual and bimanual reaching tasks. The eye of the lesion is exactly on the neuron that gets activated whenever the arm reached its target.

In addition, we tested the model performance in chronic and acute stroke cases. In chronic stroke, the MC was trained with lesion and then tested. This could be equated to a condition where therapy was provided a few months after the incidence of stroke. While in acute stroke condition, the MC was trained normally and tested right after introducing the lesion without any further retraining. This could be analogous to a condition where therapy was provided soon after the incidence of stroke.

Upon analysis, for chronic stroke, both unimanual and bimanual conditions of the task completion is observed. It is found that for lesion sizes ranging between 1 × 1 and 5 × 5, bimanual training proved to be more beneficial in terms of reduced reaching errors of the paretic arm, whereas lesion studies beyond 5 × 5 size (6 × 6 and 7 × 7), unimanual training proved to be beneficial.

For CIMT, it is observed that the reaching error decreases as the lesion size increases when compared with bimanual condition. The value of the reaching error obtained for lesion sizes above 5 × 5, i.e. 6 × 6 and 7 × 7 is comparable to that obtained in unimanual condition. Thus, for higher lesion sizes, CIMT also proves to be beneficial when compared with bimanual condition.

**Figure 7:**
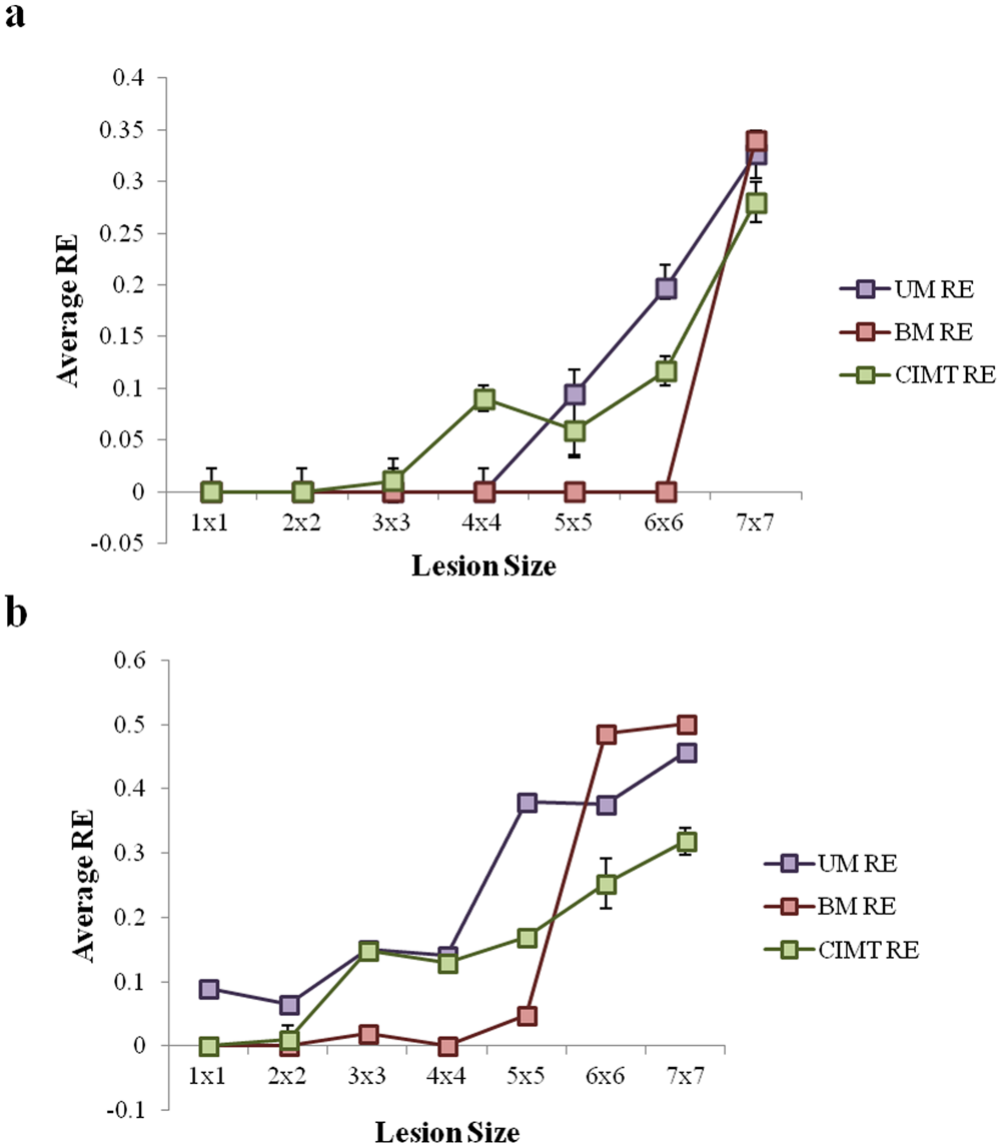
Model performance in varying lesion sizes. (**a**,**b**) The average reaching error obtained from the model under unimanual, bimanual and constraint induced training conditions plotted with respect to lesion size in acute and chronic stroke respectively.

For acute stroke, the value of reaching error is very low in bimanual condition up to a lesion size of 6 × 6. However, for 7 × 7 the error rises to a significant level (Fig. 7). The opposite pattern is observed for unimanual condition and CIMT- the value of reaching error reduces with increasing lesion size. This is similar to the trend observed in chronic stroke.

## Discussion

We propose a biologically plausible model that can perform simple bimanual reaching tasks. We consider only motor stroke affecting the upper extremities and stroke is modeled by deactivating a part of the motor cortex in the model. Since stroke is introduced at the level of motor cortex which is the hub of integration of inputs from all the other components (observed in Eq. 17), presence of stroke at this level has a direct influence on the performance of the corresponding arm, and to some extent the other arm due to coupling between the two motor cortices in the model.

Several control and stroke studies are performed using the model to compare and quantify the effects of stroke. The effect of stroke in the model on reaching behaviour of the arms is evaluated in terms of PRV and reaching error. The workspace of the arm that comprises of all reachable target locations is mapped under both stroke and post intervention paradigms. Under stroke conditions, there is an evident decrease in the number of targets the arm can reach in its workspace because of deactivation of certain neurons in the MC due to which it fails to form representations for those goal positions (Fig. 2b). Post intervention, on retraining the connections between both PC and MC that correspond to the representations lost due to lesion and MC and MN that account for specific arm configurations that were absent under stroke conditions, the ability of the arm to reach the previously unreachable targets in its workspace improved. Thus the current model accounts for the recovery of the arm after intervention and also demonstrates the potential to explain functional plasticity in the brain^30^.

The arm’s movement when it reaches the target is analyzed and the trajectories are recorded. During the initial stages of reaching the target, (between trials 1 and 20) the trajectory shows high path variability^31^. Early stage of training in the model is characterized by a low contribution from PFC and high BG contribution. The exploratory drive originating from the complex and chaotic dynamics of the STN-GPe system of the BG seems to account for high path variability in this stage. The trajectories in the later stages of training (between trials 41 and 50), become smoother with reduced path variability (refer Supplementary Material). In late stage training, the contribution from the BG is reduced, with lesser influence from the STN-GPe system, and thereby lesser exploration perhaps explaining the reduced path variability in this stage.

The velocity of the arm as it reaches the target shows the characteristic bell-shaped profile, with reduction in PRV of the arm under stroke conditions (Fig. 1c). Thus, the introduction of stroke in the model introduces a temporal delay and reduces the performance of the arm in reaching tasks as observed in classic cases of upper extremity hemiparesis^32^. These studies effectively indicate the model’s potency to simulate reaching behaviours under normal and stroke conditions.

The paper focuses on modelling and simulating the task setup of Rose *et al*. (2004) for bimanual training as a prospective rehabilitation strategy for stroke. The primary performance index analyzed in this study was the velocity profile of both the arms during reaching tasks. It was found that during the symmetric aiming task under unimanual conditions, the PRV of the non-paretic arm (left arm) was higher than that of the paretic arm (right arm). Conversely, when the arms performed the same task under bimanual conditions, the paretic arm showed an increase in its PRV and the non-paretic arm showed a significant decrease (Fig. 4). This result suggests that the non-paretic arm aids the paretic arm in moving towards its target, when the arms perform the task under bimanual conditions.

A second experiment comprising of two tasks was performed by introducing an asymmetry in the target locations. In the congruent aiming task, it was observed that although the PRV of the non-paretic arm was higher than the paretic arm in both unimanual and bimanual conditions, the PRVs of both the arms showed no significant change (Fig. 5). It may be inferred that a relatively easier performance target for the paretic arm has an insignificant influence on its PRV and hence does not contribute to any effective improvement of the arm.

In incongruent aiming, it was observed that under unimanual conditions, the non-paretic arm had a higher PRV than the paretic limb as expected. However, a surprising change was observed under bimanual conditions, where there was a significant increase in the PRV of the paretic arm accompanied by a significant decrease in the PRV of the non-paretic arm (Fig. 6). It can be deduced from these changes in the PRV, that using a more challenging task for the paretic arm would help its functional recovery (under bimanual conditions) more than using an easy one. The model is able to capture all of the abovementioned performance variations mentioned in the empirical study^29^.

Stroke rehabilitation requires a well-rounded understanding of the post-stroke effects or loss of functionality that patients suffer. Although interventions begin within the first 48 hours of stroke manifestation, only 60% of people with hemiparesis have received functional independence in activities of daily living (ADL)^33,34^. Hence, a comprehensive and customized treatment strategy that would vary from patient to patient is required for effective treatment. Progressively training components of goal-oriented tasks by reinforcing behaviour using specified learning networks or in other words, physical training has proved to be a go-to and an effective strategy implemented in stroke rehabilitation^35^ followed by other techniques that have emerged such as robotic arm training^36^, virtual reality approaches^8,37,38^ etc. The motivation behind our paper is to study these conventional training therapies using a computational model and allow the model to assess the relative merits of different therapies. To begin with, we have focused on three different rehabilitation strategies that fall under physical training therapies: Unimanual reaching tasks (URT), bimanual reaching tasks (BRT) and Constraint Induced Movement Therapy (CIMT).

CIMT is modelled by arresting the non-paretic arm (left arm in this case) in a specific configuration (this is similar to constraining the arm using a sling as observed in the experiments). The right arm (paretic arm) thus receives a constant motor cortex activity from the constrained arm modulated by the coupling strength (ε_R_ or ε_L_). Different comparison studies are performed between these rehabilitation strategies. The first study was performed by introducing *acute* stroke in the model and measuring the reaching error of the arm. Reaching error is defined as the closest distance to which the arm gets to the target location when it performs the task. This analysis is carried over different lesion sizes to assess the efficacy of the chosen rehabilitation strategy as a function of the size of lesion in the motor cortex. It is found (Fig. 7a) that until lesion size of 3 × 3 (which denotes small lesion sizes) all three rehabilitation strategies (unimanual, bimanual and CIMT) prove to be effective, and their corresponding reaching errors are zero or approximately zero. Beginning from a lesion size of 4 × 4 until 6 × 6, it can be seen that the value of reaching error is zero for bimanual but has a significant value under unimanual and CIMT conditions, thus making bimanual the most sought after strategy in this lesion bracket. Interestingly, the opposite is observed for a lesion size of 7 × 7 with bimanual reaching error being higher than the unimanual and CIMT value. However the difference in the value is not that significant.

The second study is carried out under *chronic* stroke conditions. For 1 × 1 lesion, both CIMT and bimanual training prove to be effective strategies. But from lesions of size 2 × 2 to 5 × 5, higher reaching errors in both unimanual and CIMT trainings make it a non-preferred strategy for relatively smaller lesion sizes. On the other hand, bimanual training shows a reaching error of zero or approximately zero, until a 5 × 5 lesion size but spikes suddenly at 6 × 6 thereby making it an unreliable intervention strategy at greater lesion sizes (Fig. 7b). Thus we conclude that unimanual reaching and/or CIMT are more preferred rehabilitation strategies for greater lesion sizes in the motor cortex.

Although the model gives rise to some useful predictions with respect to developing customized therapies for stroke, insufficient data on the cortical and sub-cortical connectivities still pose a challenge to simulate biologically plausible coupling within different regions of the motor network. However, the model intelligibly accounts for the inter-hemispherical connections by incorporating the coupling factor (ε) at the level of motor cortices. This is justified because the motor cortex component receives input from all the other components in the model and integrates it, dynamically influencing the performance of the system. Future work includes appending the existing model with a cerebellum component, a unified reward and value function. Since the cerebellum is involved in prediction and facilitation of fast and complicated movements^39,40^, incorporation of such a system in the model would allow these movements to be implemented in the model. Upon incorporation, the dynamics of the network needs to be modified to include the cerebellum module. Other future works include converting the PFC module into a Reinforcement Learning (RL) engine. The model would then have a hierarchical RL^41,42^ (HRL) structure with value function computation occurring at two levels – BG and PFC. The model will then be driven by the interaction between these two RL components. We also aim to bridge the gap between clinical data and modelling studies by incorporating 3D reaching tasks to facilitate the development of more realistic motor and somatosensory maps using LISSOM (Laterally Interconnected Synergistically Self Organising Maps). Thus the current model holds immense potential to be developed and used as one of the key clinical tools employed for stroke rehabilitation.

## Methods

To examine the after effect of stroke on upper extremity function, we use a cortico- basal ganglia model^31^ capable of performing simple bimanual reaching movements. In essence, the model has two semi–independent systems, each capable of driving reaching movements in a single hand. Each system has an outer loop corresponding to the cortical loop and an inner loop corresponding to the basal ganglia loop. The outer loop consists representations of two cortical modules – the *proprioceptive cortex* and the *motor cortex*, - and another module representing the spinal cord. The combined cortico-basal-ganglia system controls a simple two-link arm model. A pair of systems, corresponding to the left and right hemispherical systems, is coupled at the level of the respective motor cortices, controlling their respective arms. There exists a communication between the two motor cortices by means of a coupling factor (ϵ), reflecting inter-hemispherical connectivity. The proposed architecture is described in greater detail as follows:

### Model architecture

**Figure 8:**
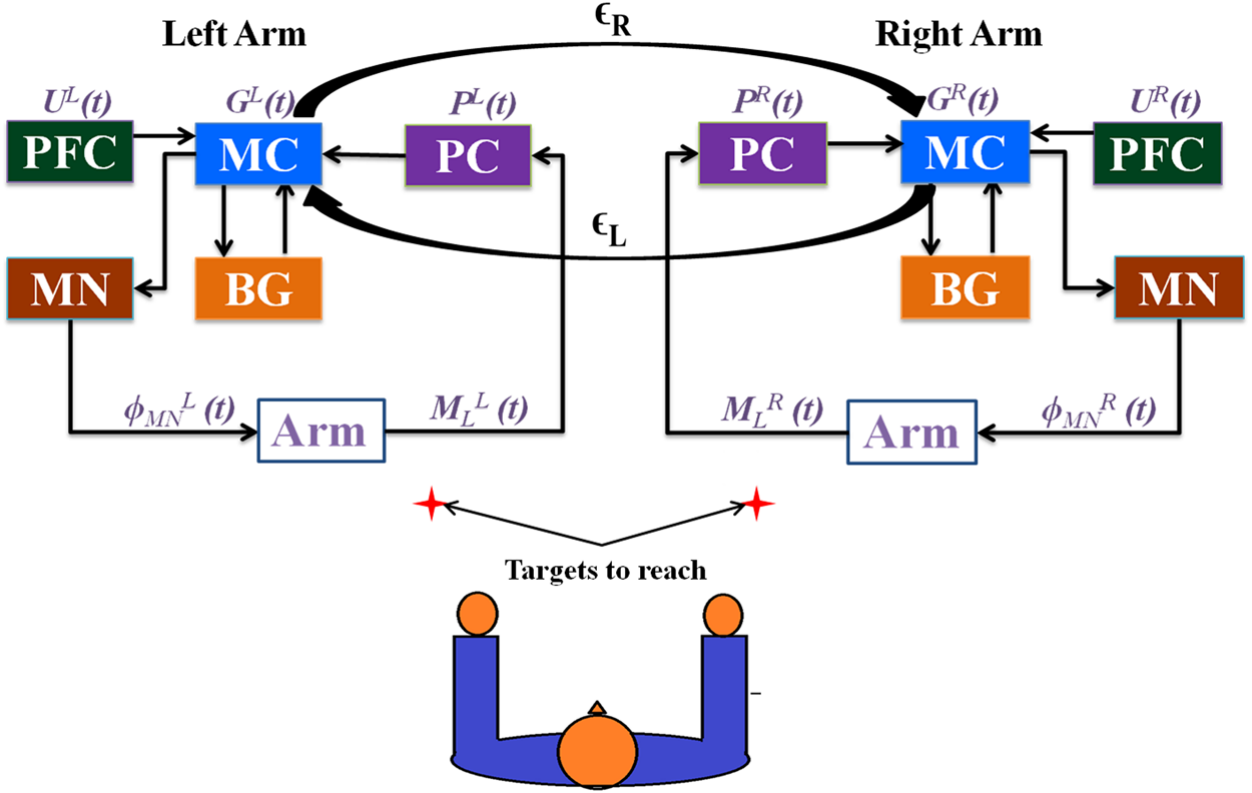
The cortico-basal ganglia model for bimanual reaching. The architecture is designed to have two loops, a sensory-motor “outer” loop and the cortico-basal ganglia “inner” loop. The motor cortex receives projections from higher frontal areas which in the model is the Prefrontal cortex. The size of the neuronal sheet used for each module in the model is 15 × 15.

The model architecture comprises of an outer sensory motor-cortical loop and an inner cortico-basal ganglia loop. The outer loop consists of the two-link kinematic arm, proprioceptive cortex (PC), motor cortex (MC) and motor neurons (MN) that innervate the two muscle pairs of the two link arm. The inner loop comprises of the basal ganglia (BG) and its components such as the striatum, globus pallidus external and internal segments (GPe and GPi), the subthalamic nucleus (STN) and the thalamus (Fig. 8).

A detailed description about every component with respect to single arm module is as follows:

#### The Sensory motor cortical loop

Arm model: The two link kinematic arm is composed of two joints where each joint is controlled by an agonist (Ag) and an antagonist (An) muscle pair. Each pair is in turn innervated by a pair of motor neurons represented in the form a four dimensional vector *ϕ*^MN^(t). The activations of the innervated muscle pairs are then used to obtain the shoulder and the elbow joint angles (θ_S/E_^JA^(t)) given by the equations:

6 $${\theta }_{S}^{JA}(t)=({\varphi }_{Ag}^{MN}(t)-{\varphi }_{An}^{MN}(t))\frac{\pi }{2}+\frac{\pi }{2}$$

7 $${\theta }_{E}^{JA}(t)=({\varphi }_{Ag}^{MN}(t)-{\varphi }_{An}^{MN}(t))\frac{\pi }{2}+\frac{\pi }{2}$$

The measures of the joint angles define the range of movements of the arm over the 2D workspace which contains a given set of targets. Following this, the lengths (*μ*^E^ and *μ*^S^) of each muscle is calculated from the joint angles by using the following equations:

8 $${\mu }_{Ag}^{S}(t)=\sqrt{{a}_{S}^{2}+{b}_{S}^{2}+2{a}_{S}{b}_{S}\,\cos ({\theta }_{S}^{JA})}$$

9 $${\mu }_{An}^{S}(t)=\sqrt{{a}_{S}^{2}+{b}_{S}^{2}-2{a}_{S}{b}_{S}\,\cos ({\theta }_{S}^{JA})}$$

10 $${\mu }_{Ag}^{E}(t)=\sqrt{{a}_{E}^{2}+{b}_{E}^{2}+2{a}_{E}{b}_{E}\,\cos ({\theta }_{E}^{JA})}$$

11 $${\mu }_{An}^{E}(t)=\sqrt{{a}_{E}^{2}+{b}_{E}^{2}-2{a}_{E}{b}_{E}\,\cos ({\theta }_{E}^{JA})}$$

A sensory (proprioceptive) map of the arm is subsequently generated from the four dimensional muscle length vector (*M*_L_ = [*μ*_Ag_^S^*μ*_An_^S^*μ*_Ag_^E^*μ*_An_^E^]). The end effector location of the arm (*X*^arm^ = [*x*_1_^arm^*x*_2_^arm^]) is computed from:

12 $${x}_{1}^{arm}=({l}_{S}-{a}_{S})\cos ({\theta }_{S}^{JA})+{l}_{E}\,\cos ({\theta }_{S}^{JA}+{\theta }_{E}^{JA})$$

13 $${x}_{2}^{arm}=({l}_{S}-{a}_{S})\sin ({\theta }_{S}^{JA})+{l}_{E}\,\sin ({\theta }_{S}^{JA}+{\theta }_{E}^{JA})$$

Formation of the sensory map: The sensory map of the arm is generated by the PC, which is modeled as a Self- Organizing Map (SOM)^39^ of size *N*_PC_ × *N*_PC._ The muscle length vector (*M*_L_(t)) is used as the feature vector to train the PC SOM. The activation of a single node *i*in the PC is given by:

14 $${P}_{i}(t)=\exp (\frac{-{\Vert {M}_{L}(t)-{W}_{PC,i}\Vert }^{2}}{{\sigma }_{PC}^{2}})$$

Formation of the motor map: The motor cortex comprises of a 2D sheet of neurons of size *N*_MC_ × *N*_MC_ and is modeled as an amalgamation of SOM and Continuous Attractor Neural Network (CANN)^43^ to account for the characteristic of low dimensional input data representation and dynamics exhibited in such cortical areas. A dynamic model like CANN is employed to facilitate the integration of multiple afferent inputs received from the PC, the BG and the Pre-Frontal Cortex (PFC).

The lateral connectivity in the CANN model is characterized by short range excitation and long range inhibition whose dynamics are defined by the weight kernel (*W*_MC_^C^) given by,

15 $${W}_{MC,i,j}^{C}={A}_{lat}^{C}\exp (\frac{-{\Vert ({i}_{MC}-{i}_{h})+({j}_{MC}-{j}_{h})\Vert }^{2}}{2{({\sigma }_{lat}^{C})}^{2}})-{K}^{C}$$

where,

*A*_lat_^C^ is the strength of the excitatory connections

*σ*_lat_^C^ is the radius of the excitatory connections and

*K*^C^ is the global inhibition constant

[*i*_MC_, *j*_MC_] are the locations of the nodes in MC, [*i*_h_, *j*_h_] corresponds to the central node.

The output from PC, a matrix of size *N*_PC_ × *N*_PC_, is converted into a vector of size *N*_PC_^2^ × 1 and given as input to the SOM part of MC for the development of motor map of the arm. There exists all-to-all connections from the muscles of the arm to PC and PC to MC. The MC SOM is trained using the standard SOM algorithm^44^. The activation of a node *i* in the SOM part of the MC is given by:

16 $${G}_{PC,i}(t)=\exp (\frac{-{\Vert P(t)-{W}_{MC,i}\Vert }^{2}}{{\sigma }_{MC}^{2}})$$

where,

*W*_MC,i_ is the weight connection between the PC and the *i*^th^ node of the SOM part of MC *σ*_MC_ is the width of the Gaussian response. The outputs from PC (G_PC_), BG (G_PC_) and PFC (G_PFC_) are then presented as input to the MC CANN. The input equation is given by:

17 $${I}_{MC}(t)={A}_{PC}{G}_{PC}(t)+{A}_{BG}{G}_{BG}(t)+{A}_{PFC}{G}_{PFC}(t)$$

where, *A*_PC_, *A*_BG_, *A*_PFC_ are the respective gains of the PC, BG and PFC networks.

With these inputs, the activation dynamics of the MC is given by:

18 $${\tau }_{MC}\frac{d{g}_{MC}}{dt}=-\,{g}_{MC}+{W}_{MC}^{C}\otimes G+{I}_{MC}$$

where *g*_MC_ is the internal state of the MC neurons.

The output MC activity (*G*(*t*)) is given by,

19 $$G(t)=\frac{{g}_{MC}^{2}}{1+(\frac{2\pi }{{N}_{MC}^{2}}){b}_{MC}\sum {g}_{MC}^{2}}$$

The MC neurons project into the four motor neurons whose activation is then given by,

20 $${\varphi }^{MN}(t)={A}_{MN}{W}_{MC\to MN}G(t)$$

To close the sensory motor loop, the MC to MN connections are trained in a supervised manner. To begin with, we give direct external input to MN, which may be considered as the desired MN activation (*φ*_D_^*MN*^(*t*)). This input will in turn produce a movement in the arm which in turn produces responses in the PC and MC in that sequence. The new MC activation presents a new input to the MN module. Now the desired MN activation (*φ*_D_^*MN*^(*t*)) and the actual MN activation (*φ*^*MN*^(*t*))) obtained after traversing over the cortical loop must be ideally the same. But in an untrained cortical loop there will be a difference. The difference between the desired and actual MN activations is used to train the connections between the MC and the MN (*W*_MC→MN_) layer:

21 $$\Delta {W}_{MC\to MN}={\eta }_{MC\to MN}({\varphi }_{D}^{MN}(t)-{\varphi }^{MN}(t))G(t)$$

where, $\Delta {W}_{MC\to MN}$ corresponds to weight updation and ${\eta }_{MC\to MN}$ is the learning rate.

Since the model is bimanual, the motor cortex of one arm is connected to the motor cortex of the other by means of a coupling factor (ϵ). Thus, a connection is established between the two hemispheres at the motor cortical level. To study upper limb hemi paretic stroke paradigms, we introduced a “lesion” in the right motor cortex by nullifying the activity of a part of the MC.

#### The Basal Ganglia

The fundamental principle of BG operation is reinforcement learning where the BG learns to choose optimal actions based on a reward feedback mechanism^45^. Hence the BG drives the arm via the motor cortex and enables the arm to reach the target. The process of action selection is guided by the presence of a value function calculated within the striatal module of the BG. Thus, the arm will learn to choose the action which brings the greatest increase in value. In the model, the value function codes for the error between the desired goal position (*X*^targ^) – provided by input from PFC – and actual end effector position (*X*^arm^) – provided by input from PC – in terms of distance. Thus the PC and PFC input are used by the striatum (value function module) to calculate the value function. The BG output then performs a stochastic hill climbing over the value function to search for the maximal value. The value is calculated by,

22 $${V}^{arm}(t)=\exp (\frac{-\,{\Vert {X}^{t{\rm{\arg }}}-{X}^{arm}\Vert }^{2}}{{\sigma }_{V}^{2}})$$

where, *σ*_V_ defines the spatial range over which the value function is sensitive for that particular target.

We then determine the value difference signal (*δ*_v_) which regulates the switching between direct and indirect pathways.

23 $${\delta }_{V}={V}^{arm}(t)-{V}^{arm}(t-1)$$

The switching happens due to the modulation of the responses from the striatal Medium Spiny Neurons (MSN). This is represented as:

24 $${y}_{D1}=\frac{1}{1+\exp (\,-\,{\lambda }_{D1}({\delta }_{V}-{t}_{D1}))}\Delta G(t)$$

25 $${y}_{D2}=\frac{1}{1+\exp (\,-\,{\lambda }_{D2}({\delta }_{V}-{t}_{D2}))}\Delta G(t)$$

where, Δ*G*(*t*) refers to the difference vector represented as difference in motor cortical activity; *y*_D1_ and *y*_D2_ represent the outputs of D1R- and D2R-expressing Medium Spiny Neurons (MSNs) respectively and *λ*_D1_ and *t*_D1_, *λ*_D2_ and *t*_D2_ are the gains and the thresholds of the direct and indirect pathway respectively. Since *λ*_D1_ = −*λ*_D2_ the dynamics of the system becomes such that when *δ*_*v*_ is positive, the direct pathway is chosen and when *δ*_*v*_ is negative, the indirect pathway is chosen.

The dynamics of the STN- GPe system influenced by *y*_D2_ is given by,

26 $${\tau }_{GPe}\frac{d{x}_{GPe}}{dt}=-\,{x}_{GPe}+{\varepsilon }_{g}\Sigma \Sigma \,{W}^{glat}{x}_{GPe}+{w}_{sg}{y}_{STN}+{y}_{D2}$$

27 $${\tau }_{STN}\frac{d{x}_{STN}}{dt}=-\,{x}_{STN}+{\varepsilon }_{s}\Sigma \Sigma {W}^{slat}{y}_{STN}-{w}_{gs}{x}_{GPe}$$

28 $${y}_{STN}=\,\tanh ({\lambda }_{STN}{x}_{STN})$$

where,

*W*^*slat*^ and *W*^*glat*^ are lateral weight connections with connection strengths *ϵ*_s_ and *ϵ*_g_ within STN and GPe respectively

*w*_*sg*_ and *w*_*gs*_ are the weight parameters that control the connection strengths.

*τ*_STN_ and *τ*_GPe_ are the respective time scales of STN and GPe.

*λ*_STN_ controls the STN output by controlling the slope of the sigmoid.

*τ*_STN_ and *τ*_GPe_ are the time scales of STN and GPe respectively.

The Gaussian neighbourhood of the lateral weight connections is given as

29 $${W}_{i,j,k,l}^{glat/slat}=\exp (-\frac{{({i}_{g/s}-{k}_{g/s})}^{2}+{({j}_{g/s}-{l}_{g/s})}^{2}}{{({\sigma }_{lat}^{g/s})}^{2}})$$

where, *σ*_lat_^g/s^ is the spread of the lateral connections respectively for the STN-GPe network.

The exploratory behaviour of the arm is attributed to the uncorrelated oscillations of the STN layer which are produced as a consequence of low striatal inputs. This is due to the formation of excitatory-inhibitory neuron pools by the STN-GPe system constituting the indirect pathway. Such excitatory-inhibitory pairs of neuronal pools are known to exhibit complex oscillations^45^.

The output signal of the direct pathway from the D1R-expressing MSNs in the striatum is integrated with the STN output in the GPi as follows:

30 $${y}_{GPi}={A}_{D1}{y}_{D1}-{A}_{D2}{y}_{STN}$$

This output from the GPi is then presented to the thalamus, also modeled as a continuous attractor neural network (CANN).

#### Representation of goal location

Goal of a movement is considered to be represented in the PFC, thereby making it the source of a motor command^46–48^. Hence, in our model the PFC is used to convey information regarding the goal position to the motor cortex. The PFC is modeled and trained as a SOM, with weights *W*_PFC_. The locations accessible by the arm in its 2D workspace are given as input feature vectors to train the SOM. The activation in the PFC corresponding to a particular target location is given by:

31 $${U}_{i}(t)=\exp (\frac{-{\Vert {X}^{t\text{arg}}(t)-{W}_{PFC,i}\Vert }^{2}}{{\sigma }_{PFC}^{2}})$$

The PFC to MC weight connections (*W*_PFC→MC_) are trained whenever the arm reaches the target. If *G*_PFC_ is the activity that is induced in the MC due to PFC activation and *G*_targ_ is the MC activity that facilitates the arm to reach its target, then the training of weights between PFC and MC is given as:

32 $$\Delta {W}_{PFC\to MC}={\eta }_{PFC\to MC}({G}^{t\text{arg}}(t)-{G}^{PFC}(t))U(t)$$

At the outset, learning occurs as the result of slow movements of the arm governed by the BG. However, in later stages, learning occurs due to fast movements dominated by the cortical loop. Hence PFC contribution increases as a function of number of trials.

### Simulation of aiming conditions

In the model, each arm had its respective target placed at locations in a manner identical to that of the experiment conducted by Rose and Winstein^29^. In the experiment, the subjects were asked to aim and hit the switches (targets) using either one arm or both arms in response to an LED signal. Based on target locations, the tasks given to the subject/model to perform were classified into:

I. Symmetric/Equidistant aiming
II. Congruent aiming
III. Incongruent aiming

All three tasks are implemented in the model, in both unimanual and bimanual conditions. In unimanual condition, the arms act independently (ϵ = 0) and in the bimanual reaching condition, they reach their respective targets simultaneously (ϵ < 0 and ϵ > 0) (Table 1). Congruent aiming task involves movement of the paretic arm to a nearby target (“near” target) with the normal arm aiming at a far off target (“far” target). The distance between the near target and the subject was half the distance between the far target and the subject. Similarly in incongruent aiming, the paretic arm had to reach the far target while the non-paretic arm moves toward a near target. These tasks are used to study the effect of reaching distance and aiming condition on the performance of the paretic arm.

**Table 1:** Parameters used in the model.

Arm Kinematics		Learning Rates				Coupling Factor			
*a*_S_	0.04	**MC** → **MN Net**				**Symmetric Aiming**			
*b*_S_	0.07	***η***_**MC→MN**_		0.1		ε _r_	−0.5	ε _l_	0.5
*a*_E_	0.03	**PFC** → **MC Net**				**Congruent Aiming**			
*b*_E_	0.08	***η***_**PFC→MC**_		0.1		ε _r_	−0.2	ε _l_	0.89
*l*_S_	0.3	λ_d1_	50	λ_d2_	−50	**Incongruent Aiming**			
*l*_E_	0.3	*t*_d1_	0	*t*_d2_	0.05	ε _r_	−0.2	ε _l_	0.8

Besides the implementation of unimanual and bimanual conditions, we have also implemented in the model, Constraint induced movement therapy (CIMT), an intervention that has shown to be effective in restoring functionality of the stroke affected limb^19,49^. The objective of this part of the modelling study is to investigate the effect of CIMT on stroke rehabilitation and understand its limitations. CIMT involves forced usage of the affected arm to perform tasks while actively restraining the unaffected arm by means of a sling or a splint. In the model, this is achieved by maintaining the unaffected arm (left) in a fixed initial configuration and allowing the paretic arm (right) to perform the task. Fixing the arm in a particular configuration ensures the imposition of a “constraint- induced” framework.

### Lesion study

To analyse the impact of the size of lesion in the MC on the performance of the paretic arm under different aiming conditions, lesions of varying size ranging from 1 × 1 to 7 × 7 are introduced in the MC. The coordinates of the center of the lesion (i_les_, j_les_) is selected such that activation of the MC near (i_les_, j_les_) places the arm at the goal location. The center of the lesion is fixed while the size is varied. This study is conducted for three aiming conditions namely unimanual, bimanual and CIMT. The arm is trained under all three conditions and is tested using symmetric targets. The lesion is centred on the (i, j)^th^ node and is expanded from a size of 1 × 1 to 7 × 7. The reaching error of the arm is computed for every lesion size as function of the distance between the actual target and end effector positions at the end of every trial.

## Supplementary information

A Cortico- Basal Ganglia Model for choosing an optimal rehabilitation strategy in Hemiparetic Stroke

## Data Availability

The code for the proposed Cortico-Basal ganglia model is available on ModelDB server (http://senselab.med.yale.edu/ModelDB/showModel.cshtml?model=245199) Access code: Stroke_code.
