# Supplementary material for: A Cortico- Basal Ganglia Model for choosing an optimal rehabilitation strategy in Hemiparetic Stroke
Source: Sci Rep. 2019 Sep 17;9:13472. doi: 10.1038/s41598-019-49670-4 (PMC6748960; doi:10.1038/s41598-019-49670-4)
Supplement: Supplementary file 1 — A Cortico- Basal Ganglia Model for choosing an optimal rehabilitation strategy in Hemiparetic Stroke [file 41598_2019_49670_MOESM1_ESM.pdf]

Supplementary Information

A Cortico- Basal Ganglia Model for choosing an optimal rehabilitation strategy in Hemiparetic Stroke

Rukhmani Narayanamurthy[1], Samyukta Jayakumar[1], Sundari Elango[1], Vignesh Muralidharan[2], and V. Srinivasa Chakravarthy[1*]

***Correspondence:** V. Srinivasa Chakravarthy: schakra@iitm.ac.in

S1. Training the outer motor cortical loop under normal conditions

The training schema of the entire cortico-basal ganglia model is shown in figure S1.

S1.1 Training the weight connections between PC and MC:

- Random activations of the agonist-antagonist muscle pairs will place the arm in 'n' different configurations with each configuration corresponding to a muscle length vector (M_L). This will then serve as feature vectors to train the SOM of the proprioceptive cortex using the standard SOM algorithm [1].
- The SOM response of the PC layer is then projected to MC SOM. Since every node in PC is connected to every node in the MC, this accounts for low dimensional representation of the sensory input to the motor cortex.

S1.2 Training the weight connections between MC and MN

- To begin with, a random activation vector is presented to the arm which subsequently activates it, thereby setting the arm in an equilibrium configuration.
- In an ideal state, the flow of this sensory information via PC to MC and then back to MN, must give rise to muscle activation equal to the random activation vector that was presented to the arm at the outset. However, since no system is ideal, the connection between MC and MN is trained by pushing the actual MN activation towards the desired activation (Section 1.3 in Methods, eqn **21**).

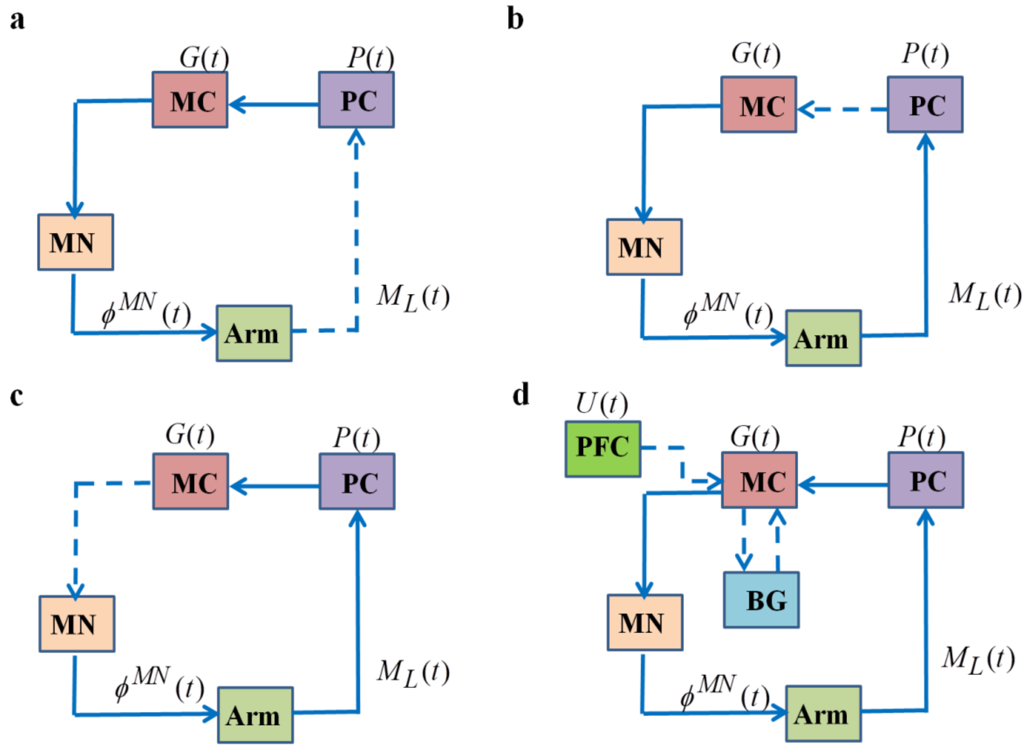

Fig. S1: The training schema in the cortico-basal ganglia model. (**a**) training the Arm to PC connections (**b**) training the PC to MC connections, closing the loop by (**c**) training the MC to MN weights. Then the BG module is introduced and the PFC to MC connections are trained (**d**). In every figure, the dashed arrows indicate the connections that are being trained.

S2. Mapping the arm configurations

The sensory motor loop is tested at the level of motor cortex to determine the range of arm movements in the workspace. A direct Gaussian current is given to the MC of size 25x25 based on the following equation:

$$I_{app} = \exp\left(-\frac{(i_{MC} - i_r)^2 + (j_{MC} - j_r)^2}{(\sigma_{lat}^{g/s})^2}\right) \tag{1}$$

where, I_{app} is the input Gaussian current, i_{MC} and j_{MC} represent the MC nodes and i_r and j_r is the random node at which the current is centred.

It is observed from figure S2(b) that, most of the positions in the 2D workspace are accessible by the arm. Also, on analogizing, we found that the activity generated probing the MC and the subsequent activity generated by signal flow via PC to MC is the same.

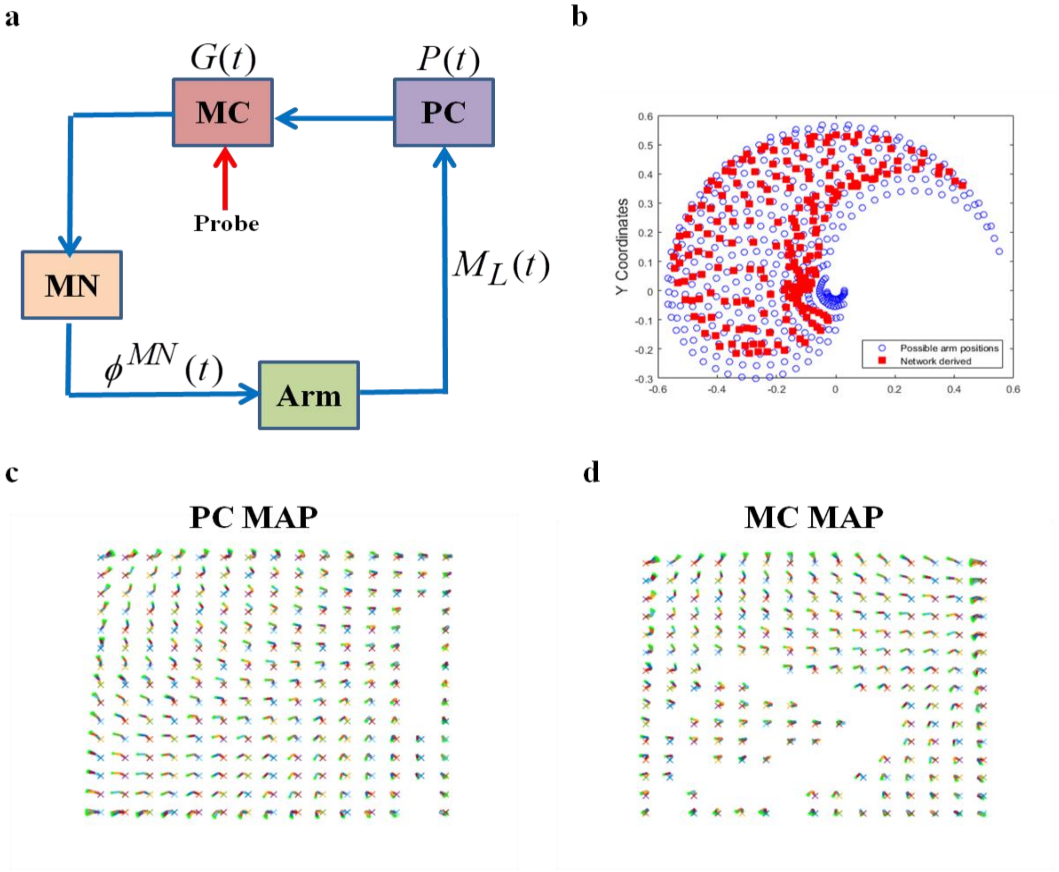

Fig. S2: Sensory and Motor Maps. (**a**) The sensory-motor loop is probed at the level of the MC. (**b**) Mapping of the end effector positions approximated by the network is compared to all possible positions in the arm workspace. (**c,d**) The joint configuration maps formed for both the PC and the MC layers respectively.

S3. Reaching movements of the arm

During early stages of learning, movements of the arm are predominantly directed by the BG output which in later stages is combined with the increasing contribution of the PFC [1]. The PFC activity that codes for the target location defines the activity that the motor cortex must evolve to in order to reach the target. The complex oscillations of the STN-GPe system enable adequate exploration of the arm in the workspace. Every time the arm moves within a distance of ‘r’ units from the target, it is considered to have accomplished its reaching task and in turn leads to training of the weight connections between PFC and MC. It is to be noted from the figure S3(a,b) that, when the PFC input to the MC and the resultant MC activity are identical, it refers to the fact that the network has learnt to approximate the activity needed to reach the target. Currently in the simulation, there are a total of 50 trials, out of which the first 30 correspond to training period of the arm and

the last 20 correspond to testing the performance of the arm. Each trial is initialized from a starting position and the goal location is kept constant throughout all trials. For every successful reach, the magnitude of the PFC contribution is increased, while training of the value function and PFC to MC connections occur in parallel. Since our model is bimanual, a trial is terminated only when both arms are successful in reaching their respective targets.

From figure S3(d), it is observed that the end effector trajectories of the arm movements, become less erratic and more smooth, indicating the decrease in hand path variability as learning progresses. This is consistent with what is experimentally observed in Stroke patients[2]. Furthermore, on analysing the velocity profile of the arm performance in control conditions, we found that it exhibits a bell-shaped profile [1]

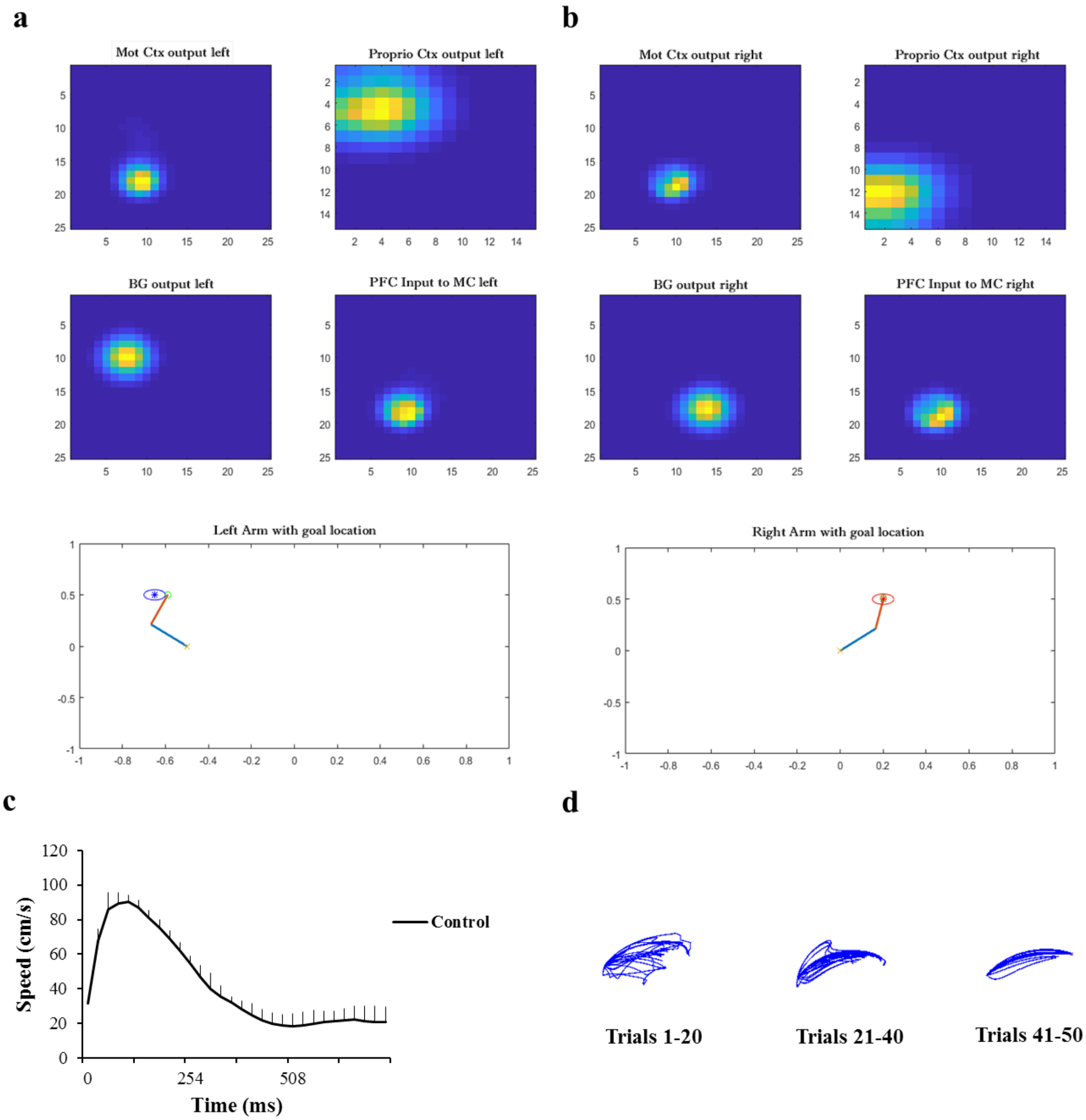

Fig. S3: Reaching behaviour in healthy controls. (**a, b**) The network output of the left and right arm while performing the reaching task independently and the activities of multiples areas in the model. (**C**) The velocity profile of the right arm during a reach; this will serve as control to compare before and after lesion introduction (**D**) The end effector trajectories obtained in the case of the control for reaching a single target across trials as the learning of the PFC to MC connections ($W_{PFC \rightarrow MC}$) takes place.

S4. Training the Value function in the Basal Ganglia

The Basal Ganglia (BG) network in the model consists of the following components – a striatum containing D1-R and D2-R expressing medium spiny neurons, a globus pallidus external (GPe), a globus pallidus internal (GPi), a subthalamic nucleus (STN) and a thalamus. Consistent with our previous models, the BG network is trained using reinforcement learning algorithm with dopamine coding for a temporal difference error signal. The value computed at every time instant t (Section 1.3 in Methods, eqn **22**), is given as the input to the BG.

The value difference signal (Section 1.3 in Methods, eqn **23**) is then carried via nigrostriatal connections to the striatum where they modulate the activity of D1-R and D2-R expressing neurons according to eqns. (**24, 25**) in Section 1.3 under Methods.

In eqns. (**24, 25**), t_{D1} and t_{D2} act as the threshold values of the Direct (DP) and Indirect pathway (IP) respectively. The DP acting via the striatum, GPi and thalamus, is responsible for movement activation, while the IP acting via the striatum, GPe, STN and the thalamus, is responsible for movement inhibition. The selection between the two pathways depends on the input to the striatum.

The value function implemented by a multilayer perceptron comprises of an input layer, a hidden layer and an output layer. PC activity at times (t) and (t-1) combined with the PFC activity serves as the input to the network and is utilized to compute the TD error. Training of the network is performed by means of error backpropagation.

The value function computed by the BG has its maximum value at the target/goal. Thus, the BG performs a stochastic hill climbing over the value function and in doing so, enables the arm to reach the goal/target. During the initial periods, the movements of the arm are governed by value gradient information; hence its behaviour tends to be more exploratory in nature. As the trials progress, the BG becomes more adept at finding the maximum of the value function thereby driving the arm to make more direct movements, whose activity is now dominated by the PFC.

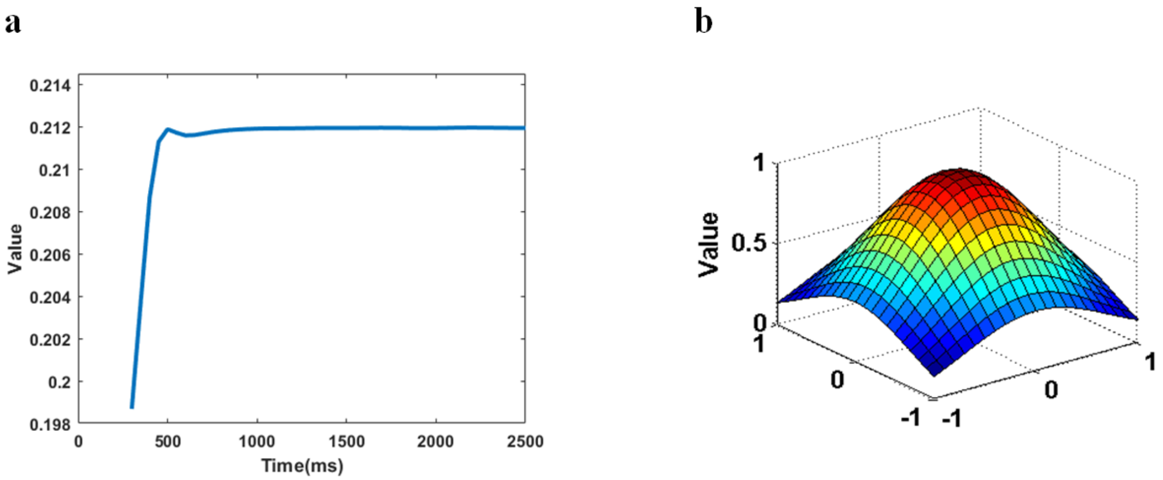

Fig. S4: Value function. (a) Value building over time. **(b)** The value function is maximum at the target location; in this case the target is at (0,0).

References

1. Muralidharan, V., et al., A Cortico-Basal Ganglia Model to Understand the Neural Dynamics of Targeted Reaching in Normal and Parkinson's Conditions, in *Computational Neuroscience Models of the Basal Ganglia*. 2018, Springer. p. 167-195.
2. Rohrer, B., et al., Movement smoothness changes during stroke recovery. Journal of Neuroscience, 2002. **22**(18): p. 8297-8304.
